# Foetal Sonographic Anogenital Distance Is Longer in Polycystic Ovary Syndrome Mothers

**DOI:** 10.3390/jcm9092863

**Published:** 2020-09-04

**Authors:** Sharon Perlman, Yoel Toledano, Zvi Kivilevitch, Nufar Halevy, Elena Rubin, Yinon Gilboa

**Affiliations:** 1 The Helen Schneider Women’s Hospital, Rabin Medical Centre, Ultrasound Unit, Zeev Jabotinsky Rd 39, Petah Tikva 49100, Israel; zvikiv@gmail.com (Z.K.); halevy.nufar@gmail.com (N.H.); elenabolot@gmail.com (E.R.); yinongilboa@gmail.com (Y.G.); 2 Sackler School of Medicine, Tel-Aviv University, Tel-Aviv 69978, Israel; toledanoyoel@gmail.com; 3 Endocrinology Clinic, The Helen Schneider Women’s Hospital, Rabin Medical Centre, Petach Tikva 49100, Israel

**Keywords:** ano-genital distance, polycystic ovary syndrome, androgen, prenatal ultrasound

## Abstract

Anogenital distance (AGD) is a biomarker for the prenatal hormonal environment. Androgen excess is a key element in polycystic ovary syndrome (PCOS). The aim of this study was to assess the sonographic foetal AGD in a population of PCOS mothers in comparison to the general population. Foetal AGD was measured prospectively by 2D ultrasound in PCOS mothers and compared to prenatal AGD nomograms. The results were interpreted regarding maternal and foetal characteristics. The mean sonographic foetal AGD centile measurement in PCOS mothers was significantly longer in comparison to the general population (86.04% ± 18.22; *p* < 0.001). Estimated foetal weight and birthweight were appropriate for gestational age and did not correlate with AGD. Sonographic foetal AGD was significantly longer in PCOS diabetic mothers and in those who conceived following assisted reproduction treatments when compared to the general population (*p* < 0.001). Our results support the role of AGD as a biomarker of the prenatal hormonal environment and provide evidence for the hyperandrogenic effect in PCOS pregnancies on foetal androgenic status and genitalia development.

## 1. Introduction

The anogenital distance (AGD) is an established anthropometric androgen-dependent parameter for genital development in animals and humans and is approximately twice as long in males than in females [1,2,3]. Animal models suggest that AGD is determined in utero [4,5]. AGD at birth reflects prenatal exposure to androgens during the masculinization programming window [6]. Several animal studies have shown that the female reproductive tract is susceptible to virilization by exogenous androgens, resulting in a longer AGD, and that exposure of the male reproductive tract to anti-androgens will result in a shorter AGD [7,8,9]. In humans, the AGD is correlated with reproductive potential in men [10,11] and women [12], with congenital anomalies of the genitalia in boys [13,14,15,16] and exposure to anti-androgens (mainly phthalate) in male neonates [17,18,19]. However, there are little data regarding the effect of the androgen milieu in utero on human foetal AGD [20,21]. In recent years we have reported the feasibility of prenatal sonographic measurement of the AGD [22] and have established foetal AGD as a quantitative biomarker of androgen exposure during the critical embryonic window of genital development [23]. These findings were confirmed in a study published by Aydin et al. in 2019 [24]. Comparisons of foetal sonographic AGD measurements obtained by Israeli and British cohorts revealed significant differences, and the authors recommended the use of population-specific normative values for accurate clinical assessments.

Mothers with polycystic ovary syndrome (PCOS), the most common endocrine disorder in women of reproductive age, have higher concentrations of circulating androgens during pregnancy [25]. Therefore, this population can serve to investigate the effects of an androgenic environment during early foetal life on in-utero development. In the present study we aimed to explore the effect of maternal PCOS on prenatal sonographic male and female foetal AGD.

## 2. Methods

A prospective pilot study was conducted over a period of 12 months.

Inclusion criteria included a well-dated pregnancy (last menstruation date or embryo transfer date confirmed by first-trimester crown rump length) and absence of associated major anomalies, including anomalies affecting the genitalia such as hypospadias, the abdominal wall and the perineum. PCOS was defined according to Rotterdam criteria; i.e., the presence of two of the following three criteria: oligo-anovulation, hyperandrogenism/hyperandrogenaemia and polycystic ovaries seen at ultrasound in the absence of all other endocrinopathies [26].

Sonographic examinations were performed with an E10 expert machine (GE Medical Systems, Kretz Ultrasound, Zipf, Austria), equipped with an abdominal RAB6-D 2–8 MHz probe.

**Figure 1:**
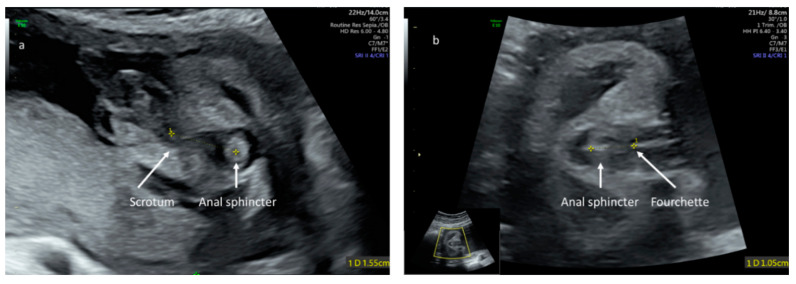
Sonographic landmarks for ano-genital distance measurement in male (**a**) and female (**b**) foetuses. In an axial view, at the level of the foetal perineum, the ano-genital distance is measured from the foetal anus to the base of the scrotum in the male or to the posterior commissure of the labia in females.

The anogenital distance was measured as described by our group [22] from the target sign, representing the foetal anus to the posterior base of the scrotum in males or the posterior commissure of the labia in females (Figure 1). Measurements were performed by experienced obstetricians with a sub-specialty in prenatal imaging (SP, YG). For each foetus, measurements were performed three times and the mean served for statistical analyses. Reproducibility of measurements for sonographic foetal AGD (inter- and intra-observer variability) was assessed in our previous study and confirmed in a following study, revealing excellent or substantial agreement [22,24].

Gender-specific centiles were calculated for the relevant gestational week according to local reference data [27].

Demographic data regarding maternal characteristics, obstetric results and neonatal outcome were retrieved from computerized medical charts.

Statistical analyses were performed with SPSS version 22.0 software (IBM Corporation, Armonk, NY, USA).

Measurements of foetal sonographic anogenital distance were analysed referring to published local normative data [22]. Differences in regard to maternal and foetal characteristics were calculated by an independent *t*-test, comparing the mean for gestational age for each gender.

The Z-score was calculated referring to mean normal value per week of gestation and gender according to published local normative data: measured AGD PCOS–mean normal value per week of gestation and gender according to normative local data [22]/normal standard deviation for each gestational week and gender.

Data were compared to local foetal AGD nomograms, and the Z-score (measured AGD PCOS–mean normal value per week of gestation and gender/normal standard deviation) for each gender was evaluated.

The study was approved by the Ethical Clinical Committee. Written informed consent was received from all patients. All subjects gave their informed consent for inclusion before participating in the study. The study was conducted in accordance with the Declaration of Helsinki, and the protocol was approved by the Ethics Committee of the Rabin Medical Center (RMC-0315-18).

## 3. Results

Altogether, 27 PCOS mothers carrying singleton foetuses (12 females and 15 males) were recruited. Maternal and foetal characteristics are presented in Table 1. The mean gestational age at measurement was 31.2 weeks ± 3 days SD (range 26–37 weeks). Mean maternal BMI was 29.36 ± 8.89 SD (range 17–36).

**Figure 2:**
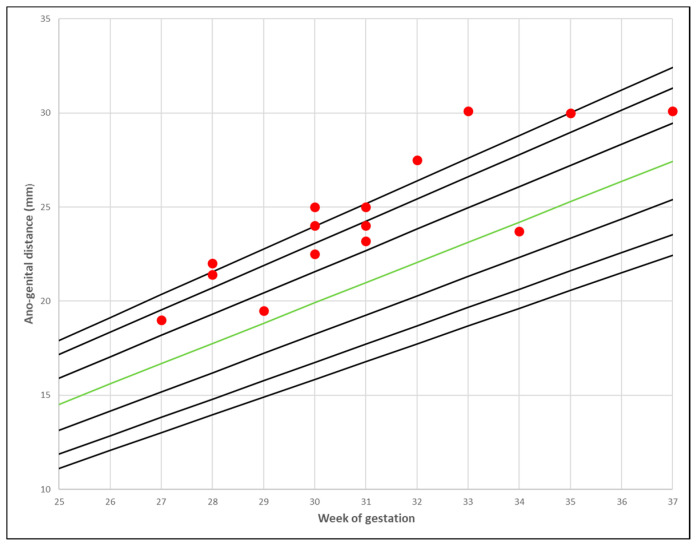
The ano-genital distance in PCOS mothers: male foetuses. The red marks represent the distribution of the measurements over the 2.5–97.5th centiles for local normal reference range. The green line represents the 50th centile [22].

**Figure 3:**
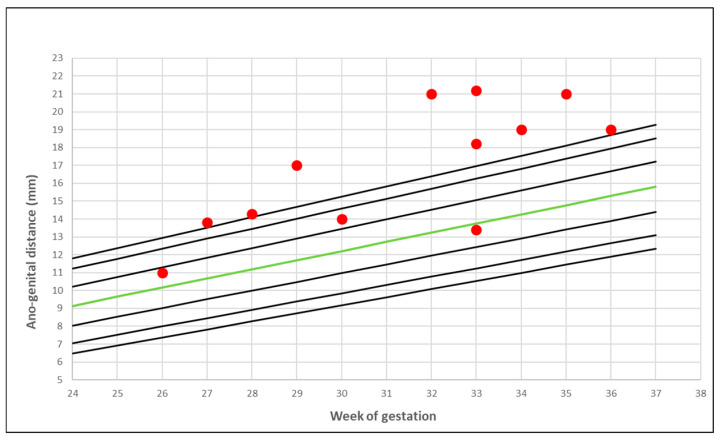
The ano-genital distance in PCOS mothers: female foetuses. The red marks represent the distribution of the measurements scattered over the 2.5–97.5th centiles for local normal reference range. The green line represents the 50th centile [22].

PCOS mothers’ foetal AGD measurements distributed over local normal reference nomograms [22] are presented in Figure 2 and Figure 3.

**Figure 4:**
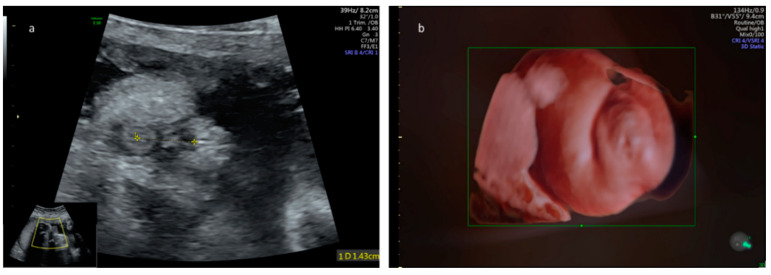
(**a**). Ano-genital distance measurements obtained in a PCOS-mothers’ female foetus at 28 weeks of gestation. The foetal anogenital distance correlates with the 97.5th percentile for gestational age [22]. (**b**). Three-dimensional surface render of the perineal region demonstrating mild clitoromegaly that could also be attributed to exposure to an intra-uterine increased level of androgens. Post-natal examination revealed normal genitalia with no evidence of virilization.

The mean sonographic foetal AGD centile measured in PCOS mothers was significantly longer in comparison to the general population (86.04% ± 18.22; *p* < 0.001) (Figure 4).

The mean Z-score with respect to the normal local reference range [22] was 2.102 (±1.85) range 0.5–9.2. The Z-score was not statistically different in males (2.035 ± 2.1950) compared to females (2.185 ± 1.3834), *p* = 0.832. Fourteen (51.9%) of the PCOS mothers were diabetic. The sonographic foetal AGD in the diabetic PCOS mothers’ foetuses was significantly longer compared to the mean local normal AGD value per week of gestation and gender (19.74 mm ± 4.8 mm vs. 15.11 mm ± 4.77 mm *p* < 0.001). However, no significant differences existed when comparing AGD measurements obtained in diabetic vs. non-diabetic PCOS mothers’ foetuses (*p* = 0.158). The mean sonographic estimated foetal weight centile and birthweight centile according to local normal reference charts were appropriate for gestational age (52.1% (±19.3) and 52.9% (±26.5), respectively) [27]. Foetal weight did not correlate with the foetal sonographic AGD (Pearson correlation 0.231, *p* = 0.313). Within the study group, 14 patients (51.9%) conceived following assisted reproduction technology (ART) (14.9% following ovulation induction and 37.0% following in vitro fertilization). The sonographic foetal AGD in the subgroup of PCOS mothers who conceived following ART was significantly longer compared to the mean local normal AGD value per week of gestation and gender (20.04 mm ± 4.42 mm vs. 15.97 mm ± 4.64 mm *p* < 0.001). However, no significant differences were demonstrated when comparing AGD measurements obtained in PCOS mothers who conceived following ART vs. those who conceived naturally (*p* = 0.377). Postnatally, all neonates were examined by paediatricians. No genital malformations were evidenced.

## 4. Discussion

Our study revealed a significantly longer sonographic foetal AGD in PCOS mothers’ foetuses and exemplified the role of AGD as a biomarker of prenatal androgen milieu. These data support previous findings [22,23] that AGD can be reliably measured in utero during the second and third trimesters of pregnancy. Assessment of AGD prior to the 20th week of gestation is not feasible as the target sign representing the foetal anus is not visualized [28,29]. In previous studies we confirmed that the correlation between AGD and male genital malformations can be successfully demonstrated in utero and that foetal sonographic AGD can serve as a reliable proxy and estimate prenatal androgen exposure and foetal reproductive programming [24]. The present pilot study aimed to assess the effect of maternal PCOS on foetal sonographic AGD. The longer foetal AGD demonstrated in our study in PCOS mothers provided indirect evidence for the increased androgenic effect in PCOS pregnancies. The characteristic metabolic disturbances of PCOS–obesity, altered lipid pattern, insulin resistance, hyperglycaemia and hyperandrogenaemia are intertwined and are all enhanced during pregnancy [30,31,32]. The source of the in utero androgen excess in PCOS mothers may be genetic, and reduced p450 aromatase activity has been demonstrated in the placentas of mothers with PCOS [33]. Although the aetiology of PCOS is unclear, prenatal exposure to androgen hormones is considered an important factor in the development of this metabolic reproductive endocrine disorder [34,35]. Anogenital distance is an established marker of androgen exposure in humans [36], and Mendiola et al. [12] reported a positive association between AGD and the presence of a greater ovarian follicular number in young women. Mira-Escolano et al. [37] examined the associations between AGD of young women and their mothers’ gynaecological characteristics before or during pregnancy and reported a positive correlation between maternal menstrual cycle irregularities and their daughters’ AGD. Wu et al. [38] and Sánchez-Ferrer et al. [39] reported a longer AGD in adult women with PCOS. Finally, Barret et al. [40] measured the AGD in 23 term female neonates born to 23 PCOS mothers and reported a significantly longer distance compared with controls. These reports suggest that the androgenic environment during early foetal life may influence the whole reproductive system development, including AGD. The results of the present study strengthen the hypotheses that PCOS pregnancies are characterised by a hyperandrogenic foetal–placental environment and that this androgenic milieu effects genitalia development in utero in both females and males. We have demonstrated that the PCOS-associated hyper androgenic effect can be evaluated by prenatal ultrasound during the third trimester of pregnancy. 

Interestingly, despite an increased incidence of hyperinsulinemia and gestational diabetes within this population, estimated foetal weight and birthweight did not correlate with AGD. This might be explained by a strict blood glucose control, since all patients were managed at a multidisciplinary clinic with endocrinology and obstetric input. The correlation between AGD and semen quality in male partners of subfertile couples was also assessed in several studies. Whilst Eisenberg and Mendiola [10,11,41], observed a strong correlation between the two parameters and considered the AGD a predictor of low sperm concentration, Parra et al. [42] reported a lack of association between AGD and semen parameters or reproductive hormone levels. In our study, there were no differences in the AGD of foetuses who were conceived following ART to those conceived spontaneously. Further research using larger sample sizes and correlation with semen parameters may shed light on the balance between paternal and maternal effects on the offspring’s AGD. The common path of abnormal maternal, placental and foetal steroidogenesis is chronic exposure of the foetus to glucose, insulin and androgens. Prenatal steroid hormones have extensive epigenetic effects on a wide range of genes. At the cellular level, sex steroids act on a variety of developmental processes including selective cell death, synaptogenesis, synaptic recruitment, pruning of synaptic spines, axon growth and neurogenesis [43]. At the clinical level, several studies provide evidence that maternal PCOS may affect the neurodevelopment of offspring, resulting in increased risk for neurodevelopmental disorders such as attention-deficit disorder and autism spectrum disorder [44,45,46]. 

Altogether, establishing AGD as a marker for in utero androgen exposure may affect the approach and treatment of PCOS mothers both prior to and during gestation, with an emphasis on modifying and optimising metabolic parameters. Lifestyle or pharmacological interventions may be considered in attempt to decrease the effect of the maternal hormonal milieu on the foetus. Since measuring human foetal androgen levels during gestation is challenging, foetal sonographic AGD has the potential to assess the efficacy of maternal metabolic modifications on the foetal hormonal milieu. This study provides support for the correlation between sonographic foetal AGD and PCOS. It calls for further research including larger and more diverse study samples, as well as using paternal and genetic data to ascertain the exact effects of androgen excess within the placental–foetal unit on the foetal genitalia, differences between genders and the potential effects of reducing maternal androgen levels.

## Figures and Tables

**Table 1:** Maternal and foetal characteristics.

Gestational Age at Measurement (Mean)	31.2 Weeks ± 3 Days SD
Maternal BMI (mean)	29.36 ± 8.89 SD
Fetal estimated weight at examination (g; percentile [27])	1852 g ± 592 g SD; 52.1% ± 19.3
Birthweight (g, percentile [27])	3110 g ± 483 g SD; 52.9% ± 26.5
Diabetes (*n*, %)	14 (51.9%)
Assisted reproduction technology (*n*, %)	14 (51.9%)
